# Analysis by Metabolomics and Transcriptomics for the Energy Metabolism Disorder and the Aryl Hydrocarbon Receptor Activation in Male Reproduction of Mice and GC-2spd Cells Exposed to PM_2.5_

**DOI:** 10.3389/fendo.2021.807374

**Published:** 2022-01-03

**Authors:** Fuquan Shi, Zhonghao Zhang, Jiankang Wang, Yimeng Wang, Jiuyang Deng, Yingfei Zeng, Peng Zou, Xi Ling, Fei Han, Jinyi Liu, Lin Ao, Jia Cao

**Affiliations:** ^1^ Key Lab of Medical Protection for Electromagnetic Radiation, Ministry of Education of China, Institute of Toxicology, College of Preventive Medicine, Army Medical University (Third Military Medical University), Chongqing, China; ^2^ School of Public Health, Shanxi Medical University, Taiyuan, China; ^3^ School of Tropical Medicine and Laboratory Medicine, Hainan Medical University, Haikou, China

**Keywords:** PM_2.5_, GC-2spd, cell proliferation, aryl hydrocarbon receptor, mitochondrial dysfunction, energy metabolism, transcriptomics, metabolomics

## Abstract

Fine particulate matter (PM_2.5_)-induced male reproductive toxicity arouses global public health concerns. However, the mechanisms of toxicity remain unclear. This study aimed to further investigate toxicity pathways by exposure to PM_2.5_ *in vitro* and *in vivo* through the application of metabolomics and transcriptomics. *In vitro*, spermatocyte-derived GC-2spd cells were treated with 0, 25, 50, 100 μg/mL PM_2.5_ for 48 h. *In vivo*, the real-world exposure of PM_2.5_ for mouse was established. Forty-five male C57BL/6 mice were exposed to filtered air, unfiltered air, and concentrated ambient PM_2.5_ in Tangshan of China for 8 weeks, respectively. The results *in vitro* and *in vivo* showed that PM_2.5_ exposure inhibited GC-2spd cell proliferation and reduced sperm motility. Mitochondrial damage was observed after PM_2.5_ treatment. Increased Humanin and MOTS-c levels and decreased mitochondrial respiratory indicated that mitochondrial function was disturbed. Furthermore, nontargeted metabolomics analysis revealed that PM_2.5_ exposure could disturb the citrate cycle (TCA cycle) and reduce amino acids and nucleotide synthesis. Mechanically, the aryl hydrocarbon receptor (AhR) pathway was activated after exposure to PM_2.5_, with a significant increase in CYP1A1 expression. Further studies showed that PM_2.5_ exposure significantly increased both intracellular and mitochondrial reactive oxygen species (ROS) and activated NRF2 antioxidative pathway. With the RNA-sequencing technique, the differentially expressed genes induced by PM_2.5_ exposure were mainly enriched in the metabolism of xenobiotics by the cytochrome P450 pathway, of which *Cyp1a1* was the most significantly changed gene. Our findings demonstrated that PM_2.5_ exposure could induce spermatocyte damage and energy metabolism disorder. The activation of the aryl hydrocarbon receptor might be involved in the mechanism of male reproductive toxicity.

## Introduction

The impact of particulate matter (PM) on health has aroused widespread public concern around the world. Fine particulate matter (PM_2.5_) is still one of the most common air pollutants in the world, especially in developing countries (1). In China, the average annual PM_2.5_ concentration in 338 cities was 10 to 86 μg/m^3^ (average: 43 μg/m^3^) in 2017, which was much higher than the recommendation of the WHO air quality guidelines (10 μg/m^3^) (2). PM_2.5_ and its combined toxic components, including transition metals, polycyclic aromatic hydrocarbons (PAHs) and water-soluble ions (WSI) can penetrate into the gas exchange area of the lungs, pass through the respiratory barrier, enter the circulatory system, diffuse to the whole body (3), and ultimately damage the respiratory system, circulatory system (4), central nervous system (5) and reproductive system (6).

Numerous epidemiology studies have shown PM_2.5_ could affect male reproduction and lead to a decrease in sperm quality (7–9). Our previous study also indicated PM_10-2.5_ exposure was inversely associated with sperm concentration in a longitudinal study of 796 Chinese college students (10). Besides the epidemiological evidence, several toxicological studies in animals revealed decreased male reproductive capacity caused by PM_2.5_. After PM_2.5_ exposure *via* intratracheal instillation, injury of testicular tissue and reduced testosterone were occurred, the integrity of blood-testis barrier was destroyed, and finally sperm quality was significantly decreased (11–13). Up to now, the mechanisms of reproductive toxicity induced by PM_2.5_ mainly focused on ROS generation, DNA damage, mitochondrial dysfunction and the disturbance of hypothalamic-pituitary-gonadal (HPG) axis. However, spermatogenesis is a complex process that requires the participation of various signal pathways, and PM_2.5_, as a complex mixture, can carry various harmful substances, for instance, PAHs (14). Numerous studies have shown that PAHs exposure might induce various adverse health effects (15–17). In male reproduction, our recent study found that PM_2.5_-bound PAHs, especially HMW PAHs decreased sperm normal morphology in the Male Reproductive Health in Chongqing College Students (MARHCS) cohort study (18). Besides, PAHs are well-known activators of the aryl hydrocarbon receptor (AhR). P. Esakky and K. Moley revealed that the PAHs of cigarette smoke condensate (CSC) showed a great effect on accelerating germ cell death through activation of AhR, which was present at all stages of spermatogenesis (19). These studies provided a possible mechanism of PM_2.5_-induced spermatogenesis damage. Nonetheless, it is still unclear whether PM_2.5_ exposure will activate AhR pathway in spermatogenesis.

Epidemiological and experimental studies have shown that mitochondria might be one of the target organelles damaged by environmental pollutants, including PM_2.5_ (20, 21). Because of the lack of protective histones and DNA repair enzymes, mitochondrial DNA is particularly vulnerable to endogenous and exogenous damage factors, such as ROS or adducts (22, 23). Meanwhile, mitochondrial are involved in various important cellular functions, including oxidative phosphorylation of ATP synthesis, mitochondrial apoptosis pathway and ROS generation, etc. Therefore, mitochondrial damage will directly cause mitochondrial dysfunction and then mediate cytotoxicity. Moreover, previous studies found PM_2.5_ can induce mitochondrial damage and activate mitochondrial apoptosis pathway in male reproduction (11, 24). Two major contributions of mitochondria in spermatogenesis involve energy generation and apoptosis (25). Because the process of spermatogenesis requires a lot of energy and the elimination of damaged spermatogenic cells, and mitochondria in sperm are arranged in the periphery of the tail microtubules to serve to energy demand for motility. It can be seen that mitochondria play a crucial part in spermatogenesis and sperm motility. However, whether PM_2.5_ exposure can disturb energy metabolism through mitochondrial damage and dysfunction in spermatogenesis is still unclear.

To better understand the effects of PM_2.5_ on male reproduction, we used GC-2spd cell line and real time whole-body PM_2.5_ exposure mouse model to investigate the effects of PM_2.5_ on mouse spermatocyte *in vitro* and *in vivo*. Then, metabolomics and transcriptomics were performed to probe into the role of energy metabolism and the aryl hydrocarbon receptor under PM_2.5_ exposure.

## Materials and Methods

### Particular Matter Suspension Preparation

Concerning the integrity of the experiment and immature technique of PM_2.5_ extraction from sampling membrane, the SRM 1648a collected in an urban area in the USA was used in the study. It was purchased from the National Institute of Standards and Technology (NIST, USA), and composed mainly of inorganic substances and organic substances with polycyclic aromatic hydrocarbons (PAHs), nitro-substituted PAHs (nitro-PAHs), polychlorinated biphenyl (PCB) congeners, and chlorinated pesticides. In addition, the particular matter has good solubility and dispersion in PBS, and a previous study indicated that the size of PM in PBS solution is from 236.43 nm to 1.98 μm (26). Thus, the PM was suspended and ultra-sonicated for 2 hours in phosphate-buffered saline (PBS) to a final concentration of 10 mg/mL. All PM_2.5_ stock solutions were stored at -80°C until biological analysis and ultrasound 30 min to avoid agglomeration of the suspended PM_2.5_ prior to treatment.

### Cell Culture and Cell Proliferation Assay

The GC-2spd cell line was purchased from American type culture collection (ATCC, CRL-2196) and cultured in Dulbecco’s modified Eagle’s medium (DMEM), supplemented with 10% FBS, 100 U/mL penicillin, and 100 μg/mL streptomycin at 37°C, 5% CO_2_. Cells were labeled with Carboxyfluorescein diacetate, succinimidyl ester (CFDA-SE; C0051, Beyotime, Shanghai, China) and then plated onto 6-well plates at a density of 1×10^5^ cells per well. PM_2.5_ stock solution (10 mg/mL) was diluted to the final concentration of 12.5, 25, 50, 100, 200 μg/mL (0, 3.91, 7.81, 15.625, 31.25, 62.5 μg/cm^2^) with complete medium. Then, cells were treated with different concentrations of PM_2.5_ suspensions. Meanwhile, the control group was added with the normal culture medium and PBS. The final concentration of PBS was less than 1%. After 24 h or 48 h, the cells were harvested, washed with PBS, and resuspended in HBSS. The fluorescence intensity was measured by a BD Accuri™ C6 flow cytometer (BD Pharmingen, San Diego, CA, United States) at an excitation wavelength of 488 nm. The FL1 detection channel was used to perform analysis, and 10,000 events were collected for each sample.

### Viability Assay for Live Cells

The cells were seeded in 6-well plates at a density of 1×10^5^ cells per well overnight. After treatment with different doses of PM_2.5_ (0, 25, 50, 100 μg/mL) for 48 h, the medium was removed and the cells were washed with PBS three times, The cells were stained with 2 μM Calcein AM (PF00008, Proteintech) and incubated for 15 min at room temperature in the dark. Images were obtained using a fluorescence microscope (Olympus IX-71, Japan) and the intensity of green fluorescent of each group was analyzed by ImageJ software.

### Animal and Treatment Protocols

Forty-five male C57BL/6 mice (6–8-week-old) were purchased from Vital River Laboratory (Beijing Vital River Laboratory Animal Technology Co., Ltd, Beijing, China). All procedures of the study were approved by the Institution Animal Care and Use Committee (IACUC). All mice were treated humanely under good laboratory conditions, and free drinking distilled water and commercial standard pellet feed were provided. After a week of adaptation, these mice (15 per group) were randomly subjected to exposure to filtered air (FA), unfiltered air (UA) and concentrated ambient PM_2.5_ (CAP) from November 18, 2019 to January 12, 2020 for a total duration of 2 months with a 12-h light/12-h dark cycle, the temperature at 24 to 26°C, and relative humidity of 40-60%. The sample size of 15 was calculated through the power analysis using an online calculator (www.stat.ubc.ca/~rollin/stats/ssize/n2.html) according to the published effect of CAP exposure on the sperm motility (27), and the statistic power is 0.90 (28). The FA-exposed mice received ambient air filtered by a high-efficient particulate air filter which was added to remove particles in the air. The UA-exposed mice were housed in the same chambers and exposed to ambient air conveyed through the pipeline. A previous study used the same apparatus had performed size classification for the particle matter in the UA chambers, and the results showed more than 90% of particle matters were less than 2.5 μm in diameter size (27). Mice exposed to concentrated ambient PM_2.5_ were performed with a PM_2.5_ concentration enrichment system (Beijing Huironghe Technology Co., Ltd, Beijing, China) located at the North China University of Science and Technology, Tangshan, China. The exposure protocol was 6 h/d, 5 days/week (only on weekdays). The real-time concentrations of PM_2.5_ in chambers and the outdoor air were constantly measured by the aerosol monitor (TSI Instrument Co., Ltd, Minneapolis, USA). The average concentrations of PM_2.5_ in the FA, UA and CAP chambers during the 2-month period were 0.39, 59.75 and 483.61 μg/m^3^, respectively. During exposure, PM_2.5_ particles were collected on quartz filter membranes (90 mm, Whatman, UK) and the membranes were weighed before and after sampling. Subsequently, analyses for water-soluble inorganic ions, metal elements and PAHs were performed in some representative membranes. Component analysis showed that PM_2.5_ particles were mainly consisted of water-soluble inorganic ions $\left(\text{N}{\text{O}}_{3}^{-},\;\text{S}{\text{O}}_{4}^{2-},\;\text{N}{\text{H}}_{4}^{+}\;\;\text{et al}.\right)$ and major metals (S, Na, Ca, Fe, K et al.). Besides, PM_2.5_ particles also contained abundant PAHs, of which benzo(b)fluoranthene (BbF) took the largest portion of PAHs, followed by fluoranthene (FLT), benzo(a)anthracene (BaA) and pyrene (PYR) (data not shown).

### Assessment of Sperm Parameter in Epididymides

To measure sperm concentration and sperm motility, the epididymides were quickly collected and cut in 800 μL Ham’s F-12 Nutrient Mixture (Gibco, USA) after the mice were anesthetized. After incubation at 37°C for 2 min, 5 μL sperm suspension was detected using a computer-assisted sperm assay (CASA; Suiplus, Beijing, China). The experimental staff were blinded to groups assignment.

### Observation of Ultrastructure of Testis and GC-2spd

Cells and testes were collected and fixed in transmission electron microscopy (TEM) fixative (Servicebio, China) at 4°C. After washing the samples using 0.1 M PB (pH 7.4) three times, the samples were post-fixed with 1% OsO_4_ in 0.1 M PB (pH 7.4) for 2 h at room temperature, then dehydrated with different proportions of ethanol. The ultrathin sections were obtained using a UC7 ultramicrotome (Leica, Germany) after Resin embedding, and then stained with 2% uranium acetate saturated alcohol solution. Finally, the ultrastructure was observed under TEM (HITACHI, Japan).

### Measurements of Oxygen Consumption Rate (OCR)

The XFp cell mito stress test was conducted to measure mitochondrial function using the XFp 8-wells Extracellular Flux Analyzer (Seahorse Bioscience, North Billerica, MA, USA) according to manufacturer’s instructions. Briefly, approximately 1,200 GC-2spd cells were seeded in the special 8-wells miniplates. After exposure to PM_2.5_ for 48 h, cells were washed with XFp assay medium containing 10 mM glucose (Agilent Technologies, 103577), 2 mM L-glutamine (Agilent Technologies, 103579) and 1 mM sodium pyruvate (Agilent Technologies, 103578). OCR was analyzed by adding oligomycin, 1.5 μM; carbonyl cyanide 4-(trifluoromethoxy) phenylhydrazone (FCCP), 2 μM and Rotenone, 0.5 μM to evaluate basal respiration, maximal respiration, spare capacity, proton leak and ATP production. And the OCR was normalized to the protein concentration of each sample.

### Measurements of Intracellular and Mitochondrial ROS

The intracellular and mitochondrial ROS production were detected using the oxidation-sensitive fluorescent probe CM-H2DCFDA (Invitrogen, USA) and MitoSOX Red (Invitrogen, USA), respectively. According to the manufacturer’s instructions, the GC-2spd cells were seeded in 6-wells plates at a density of 1×10^5^ cells per well. After treatment with 0, 25, 50, 100 μg/mL PM_2.5_ for 48 h, the cells were harvested and washed in PBS, followed by incubation with 10 μM CM-H2DCFDA for 30 min at 37°C and 5 μM MitoSOX Red for 10 min at 37°C, respectively. The mean fluorescence intensity was measured by a BD Accuri™ C6 flow cytometer (BD Pharmingen, San Diego, CA, United States) and analyzed using FlowJo software (Tree Star, Inc., San Carlos, CA, USA).

### Evaluations of Humanin and MOTS-c Levels

The levels of Humanin and MOTS-c in GC-2spd cells were measured using commercially available enzyme-linked immunosorbent assay (ELISA) kits (Shanghai Enzyme-linked Biotechnology Co., Ltd, Shanghai, China) according to the manufacturer’s directions. The minimum detectable doses were both typically less than 0.1 ng/mL. The intra-assay CV (%) and inter-assay CV (%) were less than 15%, respectively. The final levels of Humanin and MOTS-c were normalized to total protein.

### RNA Extraction and Quantitative Reverse‐Transcription Polymerase Chain Reaction (qRT‐PCR)

The total RNA from the cells and testes was isolated using TRIzol reagent (Invitrogen, Carlsbad, CA, USA) and converted to cDNA using RevertAid Master Mix (ThermoFisher, USA). PCR was performed on a CFX Real‐Time System (Bio‐Rad Laboratories Inc.) using GoTaq qPCR Master Mix (Promega, USA) and specific primers. The PCR amplification schedule started with 95°C for 10 min, followed by 45 cycles of 95°C for 30 s and 60°C for 30 s. The cycle threshold (Ct) values were recorded, and relative expression of the target gene was calculated through the 2^-△△Ct^ method. The primers (Sangon Biotech, Shanghai, China) were used as follows: ACTB (forward 5′‐GTGACGTTGACATCCGTAAAGA‐3′, reverse 5′‐ GCCGGACTCATCGTACTCC‐3′); *Cyp1a1* (forward 5′‐GGCCACTTTGACCCTTACAA‐3′, reverse 5′‐ CAGGTAACGGAGGACAGGAA‐3′).

### Western Blot

Cells and testicular tissues were collected and lysed with a cell lysis buffer (Beyotime, Shanghai, China). Subsequently, 40-60 μg protein was resolved by 12% SDS-PAGE and then transferred to polyvinylidene difluoride (PVDF) membranes (Merck Millipore, Burlington MA, USA) for western blot analysis. Blots were blocked with 3% bovine serum albumin (BSA) in 0.15% Tris-Buffered Saline with Tween (0.15% TBST), and then probed with primary antibodies overnight at 4°C. The primary antibodies were used as follows: AHR Polyclonal Antibody (17840-1-AP, Proteintech); CYP1A1 Polyclonal Antibody (13241-1-AP, Proteintech); NRF2 Polyclonal Antibody (16396-1-AP, Proteintech); KEAP1 Polyclonal Antibody (10503-2-AP, Proteintech); β-actin Rabbit Antibody (4970S, Cell Signaling Technology, USA); Lamin B1 Rabbit Antibody (13435S, Cell Signaling Technology, USA). PCNA mouse Antibody (sc-56, Santa Cruz Biotechnology, USA). After three washes with 0.15% TBST, blots were treated with horseradish peroxidase-conjugated secondary antibodies (ab205718 and ab205719, Abcom, USA). Finally, protein bands were visualized using the enhanced chemiluminescence system, and the signals of blots were measured with ImageJ software. β-actin and Lamin B1 were utilized as the internal control for the normalization of the expression.

### Extraction of Cytoplasmic and Nuclear Protein

To evaluate AhR and NRF2 nuclear translocation by Western blotting, cytoplasmic and nuclear proteins were separately extracted using Qproteome Nuclear Protein Kit (QIAGEN, Germany) following its instructions. In brief, after exposure to PM_2.5_ for 48 h, cells were washed twice with ice-cold PBS, harvested using cell-scraper and centrifuged to remove supernatant. The cell pellets were resuspended in lysis buffer NL (supplemented with protease inhibitor solution and 0.1 M DTT) and incubated for 15 min on ice. Then, the detergent solution NP was added, and the cell suspensions were centrifuged for 5 min at 10,000 x g after vortex. The supernatants (cytoplasmic fraction) were transferred into new microcentrifuge tubes and stored at –80°C. The remaining pellets which contain cell nuclei were resuspended in nuclear protein lysis buffer NL (supplemented with protease inhibitor solution and 0.1 M DTT). After vortex and centrifugation, the nuclear pellets were obtained and resuspended in extraction buffer NX1 (supplemented with protease inhibitor solution). Finally, after incubation for 30 min and centrifugation at 12,000 x g for 10 min, the supernatants (nuclear fraction) were transferred into new tubes and stored at –80°C until use.

### Metabolite Extraction, Dection, and Analysis

The GC-2spd cells with 80% confluency in a 10 cm dish were exposed to PM_2.5_ (0, 100 μg/mL) for 48 h, and the medium was discarded followed by washing the cells three times with ice-cold PBS. Then the cells were harvested after adding trypsin and washed three times with ice-cold PBS, frozen and thawed with liquid nitrogen for 3 times, finally sonicated for 10 min in ice-water bath. 50 μL of homogenate was used to measure protein concentration. Then 600 μL acetonitrile: methanol = 1: 1 was added to the rest part and transferred to 2mL EP tube. After 30 s vortex, the samples were incubated at -40°C for 1 h and centrifuged at 12,000 rpm for 15 min at 4°C. 660 μL supernatant was transferred to an EP tube and dried in a vacuum concentrator. Then acetonitrile: methanol: water = 2: 2: 1, with isotopically-labelled internal standard mixture was added in proportion. After 30 s vortex, the samples were sonicated for 10 min in ice-water bath, centrifuged at 12,000 rpm for 15 min at 4°C. The resulting supernatant was transferred to a fresh glass vial for analysis, and the quality control (QC) sample was prepared by mixing an equal aliquot of the supernatants from all of the samples.

LC-MS/MS analyses were conducted by using an UHPLC system (Vanquish, Thermo Fisher Scientific) with a UPLC BEH Amide column (2.1 mm × 100 mm, 1.7 μm) coupled to Q Exactive HFX mass spectrometer (Orbitrap MS, Thermo). The mobile phase consisted of 25 mmol/L ammonium acetate and 25 ammonia hydroxide in water (pH = 9.75) (A) and acetonitrile (B). The auto-sampler temperature was 4°C, and the injection volume was 3 μL. The QE HFX mass spectrometer was used for its ability to acquire MS/MS spectra on information-dependent acquisition (IDA) mode in the control of the acquisition software (Xcalibur, Thermo). In this mode, the acquisition software continuously evaluated the full scan MS spectrum. The ESI source conditions were set as follows: sheath gas flow rate as 30 Arb, Aux gas flow rate as 25 Arb, capillary temperature 350°C, full MS resolution as 60000, MS/MS resolution as 7500, collision energy as 10/30/60 in NCE mode, spray Voltage as 3.6 kV (positive) or -3.2 kV (negative), respectively. For the raw data, we converted it to the mzXML format using ProteoWizard and processed using an in-house program, which was developed by R and based on XCMS, for peak detection, extraction, alignment, and integration. Then an in-house MS2 database (BiotreeDB) was applied in metabolite annotation. The cutoff for annotation was set at 0.3.

The final dataset containing the information of peak number, sample name and normalized peak area was imported to SIMCA16.0.2 software package (Sartorius Ste dim Data Analytics AB, Umea, Sweden) for principal component analysis (PCA) and orthogonal projections to latent structures-discriminate analysis (OPLS-DA). In addition, a 7-fold cross validation was performed to estimate the robustness and predictive ability of our model after 200 times permutations. According to OPLS-DA, a loading plot was constructed to show the contribution of variables to differences between the two groups. Furthermore, the value of variable importance in the projection (VIP) of the first principal component in OPLS-DA analysis was obtained, which summarized the contribution of each variable to the model. The metabolites with VIP>1 and p<0.05 (student’s t-test) were considered as significantly changed metabolites. In addition, commercial databases including KEGG (http://www.genome.jp/kegg/) and MetaboAnalyst (http://www.metaboanalyst.ca/) were used for pathway enrichment analysis.

### Transcriptome Analysis

Transcriptome analysis was applied to investigate global RNA changes after PM_2.5_ exposure. The total RNA from the GC-2spd cells was isolated using TRIzol reagent (Invitrogen, Carlsbad, CA, USA). The RNA quantification and qualification were assessed using the NanoPhotometer^®^ spectrophotometer (IMPLEN, CA, USA) and RNA Nano 6000 Assay Kit of the Bioanalyzer 2100 system (Agilent Technologies, CA, USA). A total amount of 1 µg RNA per sample was used for the RNA sample preparations. Sequencing libraries were structured with NEBNext^®^ UltraTM RNA Library Prep Kit for Illumina^®^ (NEB, USA) according to the manufacturer’s recommendations. After cluster generation, the library preparations were sequenced on an Illumina Novaseq platform and 150 bp paired-end reads were generated. To ensure the quality and reliability of data analysis, the raw data were filtered, and all the downstream analyses were based on clean data with high quality. Next, we selected Hisat2 as the mapping tool to the reference genome. FPKM was calculated based on the length of the gene *via* featureCounts v1.5.0-p3. Differential expression analysis of two groups was proceeded with the DESeq2 R package. Genes with an adjusted p-value <0.05 found by DESeq2 were considered as differentially expressed genes (DEGs). Gene Set Enrichment Analysis (GSEA; http://www.broadinstitute.org/gsea/index.jsp) was conducted based on all changed genes.

### Statistical Analyses

Quantitative data are expressed as the means ± standard deviation (SD) of three experiments unless noted otherwise. Statistical comparisons were performed by *t*-test and one-way ANOVA analysis of variance followed by the Dunnett’s multiple comparison test. All subjective analyses were performed by individuals blinded to treatment groups. Statistical analysis was conducted using SPSS (version 26.0) and GraphPad Prism (version 8.4.0; GraphPad Software). A *P*-value < 0.05 was considered significance level.

## Results

### PM_2.5_ Inhibits Cell Proliferation in GC-2spd and Reduces Sperm Motility in Testes

**Figure 1:**
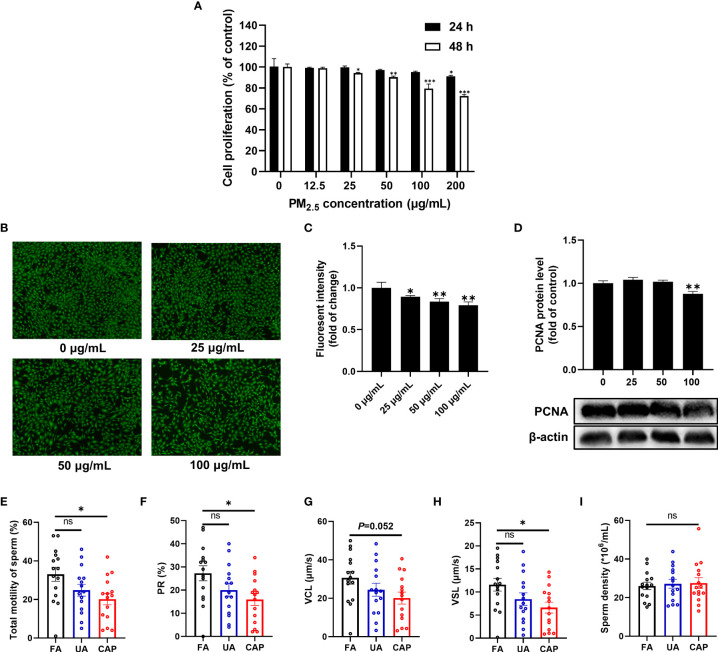
PM_2.5_ inhibits cell proliferation in GC-2spd and reduces sperm motility in testes. **(A)** Cells were treated with different concentrations of PM_2.5_ (0, 12.5, 25, 50, 100, 200 μg/mL) for 24 or 48 h. Subsequently, cell proliferation was measured using CFDA-SE Cell Proliferation Assay and Tracking Kit. **(B)** Cells were treated with PM_2.5_ (0, 25, 50, 100 μg/mL) for 48 h, and live cells were determined with Calcein AM staining followed by fluorescence microscope (100 X). **(C)** The intensity of green fluorescent was analyzed by ImageJ software. **(D)** The expression of PCNA was detected by western blot analysis, and the bar graph shows the quantification of PCNA. Values are presented as means ± SD (n=3). Sperm motility and sperm density changes of mice testes after exposure to PM_2.5_. **(E)** Total motility of sperm; **(F)** PR% (progressive motility) of sperm; **(G)** VCL (curvilinear velocity) of sperm; **(H)** VSL (straight-line velocity) of sperm; **(I)** Sperm density. Data are presented as means ± SEM (n = 15). *P < 0.05, **P < 0.01, and ***P < 0.001. ns, not significant.

The effect of PM_2.5_ exposure on GC-2spd was examined firstly. As shown in **Figure 1A**, PM_2.5_ exposure can significantly affect cell proliferation in a dose and time-dependent manner. The cell proliferation was significantly reduced in the doses of 200 μg/mL (equal to 62.5 μg/cm^2^) after treatment for 24 h. When the exposure time was extended to 48 h, cell proliferation was significantly inhibited from the dose of 25 μg/mL (equal to 7.81 μg/cm^2^), and the inhibition ratio was about 20% in the dose of 100 μg/mL (equal to 31.25 μg/cm^2^). In live cell staining experiment, the percentage of viable cells was about 80% when exposed to PM_2.5_ (100 μg/mL) for 48 h (**Figures 1B, C**). Besides, the protein level of proliferating cell nuclear antigen (PCNA), a cell proliferation marker decreased markedly after PM_2.5_ treatment for 48 h (**Figure 1D**). In consideration of the numbers and structures of viable cells, the largest dose of 100 μg/mL was adopted in the subsequent experiment.

In the animal study, we examined the sperm quality of mice testes using the whole-body PM_2.5_ exposure mouse model. As shown in **Figure 1E**, exposure to CAP significantly decreased the total motility of sperm compared to the FA group. Likewise, significant changes were also observed in the motility parameters like PR% (progressive (motility), *P*<0.05) and VSL (straight-line velocity, *P*<0.05) (**Figures 1F–H**). However, no obvious change in sperm density was detected between FA and PM_2.5_ groups (**Figure 1I**).

### PM_2.5_ Induces Mitochondrial Damage and Dysfunction in GC-2spd and Testes

**Figure 2:**
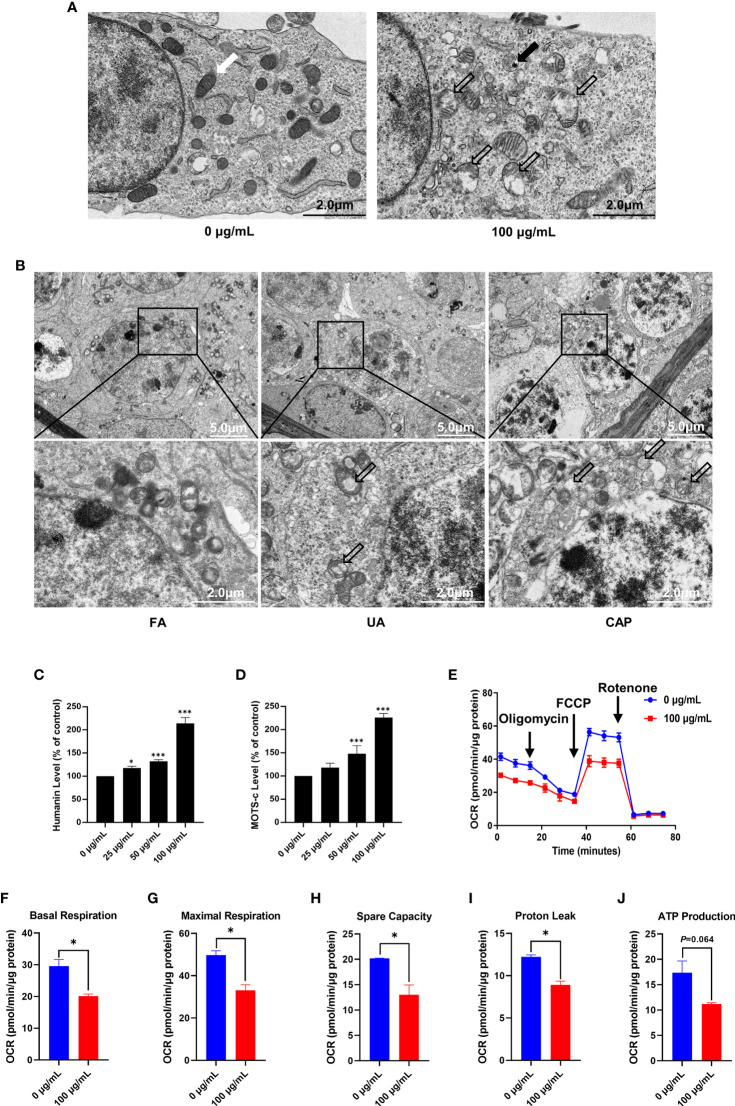
PM_2.5_ induces mitochondrial damage and dysfunction in GC-2spd and testes. **(A)** The ultrastructure of GC-2spd was observed by transmission electron microscopy after treatment with 0 or 100 μg/mL PM_2.5_. The white arrow indicates normal mitochondria; the thick black arrow represents electron-dense PM_2.5_ in the autolysosome; the hollow arrows represent swollen and cavitation of mitochondria. **(B)** The ultrastructure of spermatocyte in mice testes after exposure to PM_2.5_. The hollow arrows represent swollen and cavitation of mitochondria. The humanin **(C)** and MOTS-c **(D)** levels were measured using ELISA kit. **(E)** The oxygen consumption rate (OCR) was measured in GC-2spd using the Seahorse XFp Cell Mito Stress test after injection of the mitochondrial ETC inhibitors oligomycin (an inhibitor of complex V), FCCP (which uncouples the proton gradient), and rotenone (an inhibitor of complex I). The basal respiration **(F)**, maximal respiration **(G)**, spare capacity **(H),** proton leak **(I)** and ATP production **(J)** were evaluated. *P < 0.05 and ***P < 0.001. Values are presented as means ± SD (n = 3).

The cellular ultrastructure was observed after PM_2.5_ treatment for 48 h by using transmission electron microscopy. In the control group (0 μg/mL), the mitochondria were abundant and showed normal shape. The structure of mitochondrial cristae was also clear and intact. While the ultrastructure of GC-2spd in 100 μg/mL PM_2.5_ group displayed mitochondria swelling, cristae disruption, and even vacuolization (**Figure 2A**). In addition, aggregated PM_2.5_ was found in the autolysosome (**Figure 2A**). Furthermore, the ultrastructure of mice testes showed that the spermatocyte displayed mitochondria swelling and cristae disruption in UA and CAP groups (**Figure 2B**).

Humanin and MOTS-c belong to mitochondrial derived peptide (MDP) which is a polypeptide encoded by short open reading frames in mitochondrial DNA (29). MDP is another type of retrograde signaling molecule with biological activity in response to cellular stress, and can reflect mitochondrial function to some extent (30). The ELISA kit revealed that exposure to PM_2.5_ significantly increased humanin and MOTS-c level in a dose-dependent manner, and both humanin and MOTS-c levels were more than 2 times higher than control in the 100 μg/mL (**Figures 2C, D**). To provide a more comprehensive evaluation of mitochondrial function in GC-2spd, we examined the effects of PM_2.5_ on oxidative phosphorylation and oxygen consumption rate (OCR) of cells using the Seahorse Mito stress test (**Figure 2E**). Compared with the control group (0 μg/mL), PM_2.5_ exposure significantly decreased basal respiration, maximal respiration, spare capacity and proton leak (**Figures 2F–I**). ATP production was also reduced but no significant effect was found (**Figure 2J**).

### PM_2.5_ Activates AhR-CYP1A1 Pathway in GC-2spd and Testes

**Figure 3:**
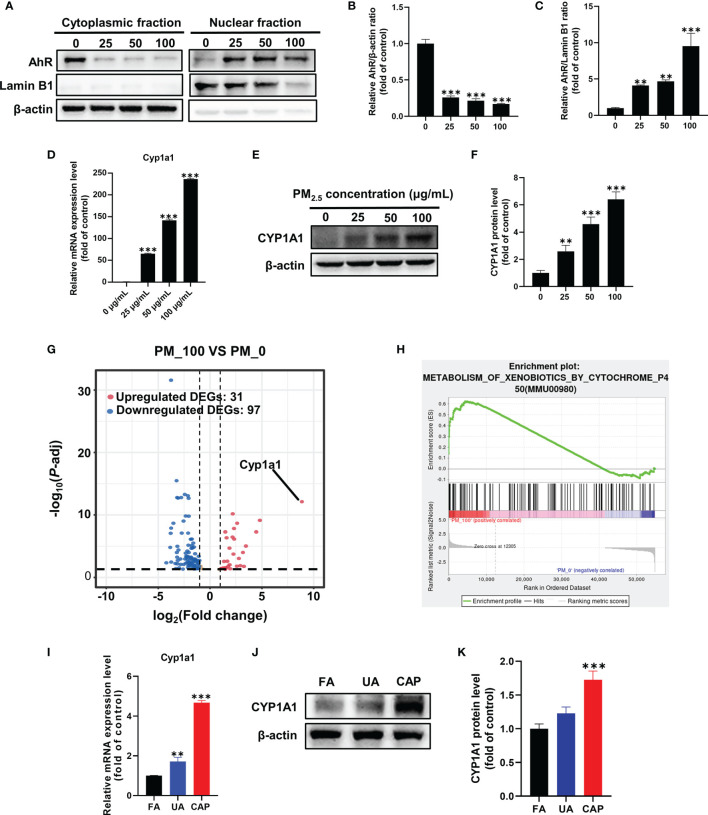
PM_2.5_ activates AhR-CYP1A1 pathway in GC-2spd and testes. GC-2spd cells were treated with PM_2.5_ (0, 25, 50, 100 μg/mL) for 48 h and the cytoplasmic and nuclear fractions were separately extracted. **(A)** The nuclear translocation of AhR was examined by western blot analysis. The bar graphs show the quantifications of the indicated proteins, of which β‐actin **(B)** and Lamin B1 **(C)** were used as internal controls of cytoplasmic or nuclear protein, respectively. The mRNA **(D)** and protein **(E)** expressions of CYP1A1 were measured in GC-2spd using qRT-PCR and western blotting, respectively. **(F)** The bar graph shows the quantification of CYP1A1 protein. The Volcano Plot **(G)** and GSEA result **(H)** were represented after transcriptome analysis. The mRNA **(I)** and protein **(J)** expressions of CYP1A1 in testes were measured using qRT-PCR and western blotting, respectively. **(K)** The bar graph shows the quantification of CYP1A1 protein. **P < 0.01 and ***P < 0.001. Values are presented as means ± SD (n = 3).

The SRM 1648a PM_2.5_ contains abundant PAHs, and PAHs are well-known activators of the aryl hydrocarbon receptor (AhR) which is a ligand-activated transcription factor. Thus, we assessed the possible implication of AhR in our study. The status of AhR nuclear translocation was firstly examined using Western blotting by separately extracting cytoplasmic and nuclear protein. As shown in **Figure 3A**, the expression of AhR protein was significantly reduced in the cytoplasmic fraction even in a low concentration of PM_2.5_, nevertheless it was obviously increased in the nuclear fraction comparing with the control group (**Figures 3B, C**). These results prompted PM_2.5_-associated PAHs could bind to and activate AhR. CYP1A1, a xenobiotic-metabolizing phase I enzyme, is the target of the AhR. In our study, the *Cyp1a1* mRNA expression was significantly increased, more than 200 times higher than that in the control group (**Figure 3D**). Similarly, CYP1A1 protein expression was remarkably improved in a dose-dependent by PM_2.5_ (about 6-fold increase in 100 μg/mL) (**Figures 3E, F**).

To explore the molecular mechanism by which PM_2.5_ exposure-induced mitochondrial damage, we conducted RNA-sequencing analysis to investigate the changed genes and pathways. A total of 128 differentially expressed genes (DEGs) (31 upregulated DEGs and 97 downregulated DEGs) were identified (fold change >1 and *P*<0.05) in contrast with the control group, and the fold change of these DEGs was visualized by Volcano plot (**Figure 3G**). It was worth noting that *Cyp1a1* was the most significantly changed gene (468-fold increase in 100 μg/mL). To further investigate which signaling pathway was regulated by PM_2.5_ exposure, all changed genes were further assessed *via* gene set enrichment analysis (GSEA). Consistently, GSEA showed that up-regulated genes were significantly enriched in the metabolism of xenobiotics by the cytochrome P450 pathway (**Figure 3H**). The RNA-Seq analysis confirmed that AhR-CYP1A1 pathway played a key role in mitochondrial damage caused by PM_2.5_ exposure. Meanwhile, we examined the expression of CYP1A1 in mice testes. The results found that PM_2.5_ exposure significantly increased the expressions of CYP1A1 in the mRNA and protein levels (**Figures 3I–K**).

### PM_2.5_ Induces ROS Production and Activates NRF2 Antioxidative Pathway in GC-2spd and Testes

**Figure 4:**
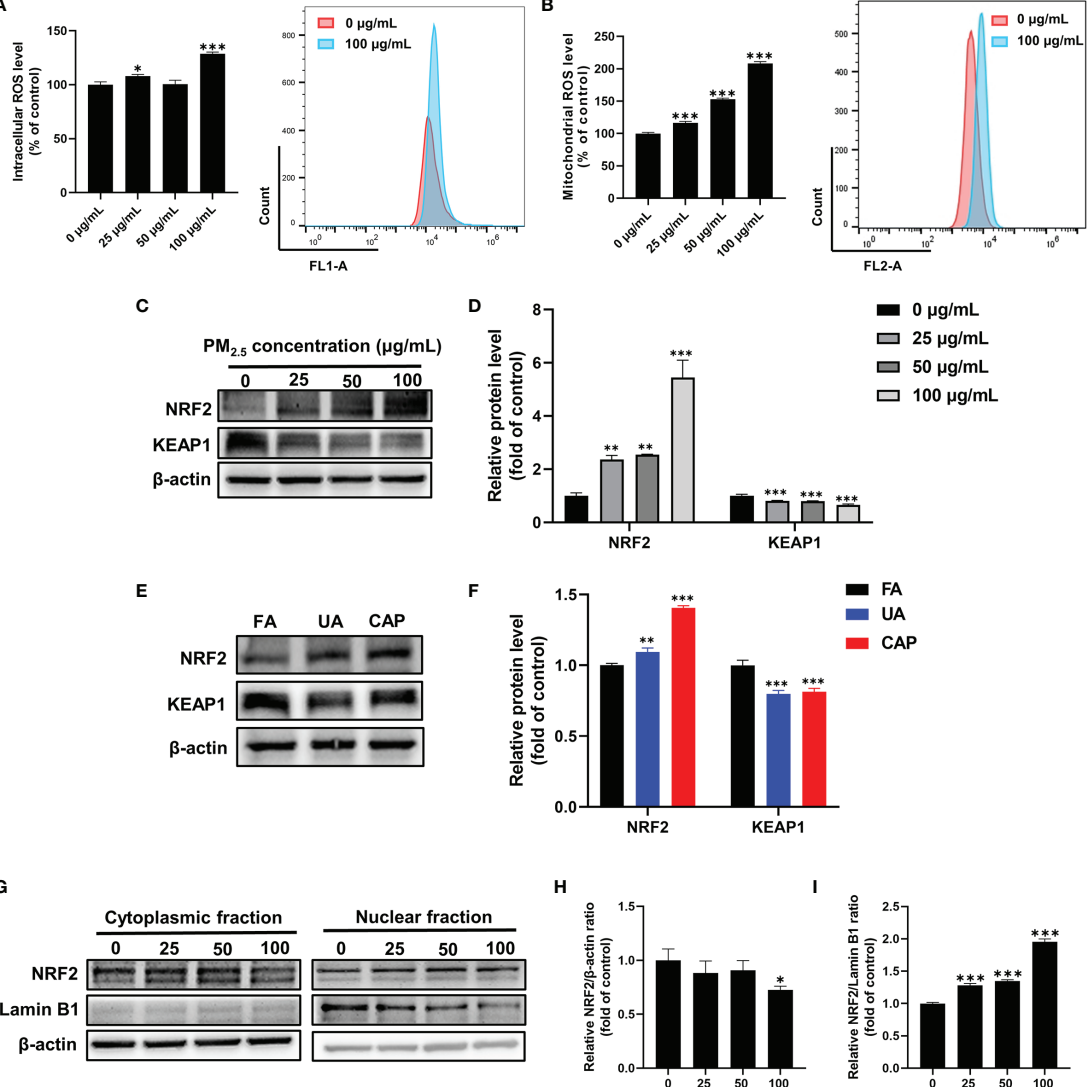
PM_2.5_ induces ROS production and activates NRF2 antioxidative pathway in GC-2spd. GC-2spd cells were treated with PM_2.5_ (0, 25, 50, 100 μg/mL) for 48 h and the intracellular **(A)** and mitochondrial **(B)** ROS were measured by flow cytometry. **(C)** The protein expressions of NRF2 and KEAP1 were measured in GC-2spd by western blot analysis. **(D)** The bar graph shows the quantifications of NRF2 and KEAP1 in GC-2spd. **(E)** The protein expressions of NRF2 and KEAP1 were measured in testes by western blot analysis in mice testes. **(F)** The bar graph shows the quantifications of NRF2 and KEAP1 in mice testes. **(G)** The cytoplasmic and nuclear fractions were separately extracted and the nuclear translocation of NRF2 was examined by western blot analysis. The bar graphs show the quantifications of the indicated proteins, of which β‐actin **(H)** and Lamin B1 **(I)** were used as internal controls of cytoplasmic or nuclear protein, respectively. *P < 0.05, **P < 0.01, and ***P < 0.001. Values are presented as means ± SD (n = 3).

Several studies indicate that PM and AhR-mediated induction of CYP1A1 lead to excessive production of ROS, which contributes to oxidative stress (16). Therefore, we examined mitochondrial ROS and intracellular ROS in GC-2spd after PM_2.5_ exposure for 48 h using flow cytometry. As shown in **Figures 4A, B**, the mitochondrial ROS was significantly increased with the increase in doses (approximately 2-fold higher in 100 μg/mL) compared to the control group. Similarly, the intracellular ROS generation was also increased in a higher concentration of PM_2.5_. NRF2, as an antioxidative transcription factor, can interact with AhR pathway. It is bound to the KEAP1 in the cytoplasm and activated by stress signals, such as ROS. After dissociation of KEAP1, NRF2 transfers to nuclear and binds to antioxidant response elements (AREs) (31). To know whether PM_2.5_ is capable of activating the NRF2 antioxidative pathway, we next assessed the protein expressions of NRF2 and KEAP1. Western blotting showed the total NRF2 protein was significantly increased accompanied by a decrease in KEAP1 protein expression in GC-2spd (**Figures 4C, D**). Moreover, comparable results were found in mice testes (**Figures 4E, F**). The activation of NRF2 involves the process of cytoplasmic-to-nuclear translocation, thus the NRF2 nuclear translocation was confirmed by separately extracting cytoplasmic and nuclear protein. As shown in **Figures 4G–I**, NRF2 protein expression in the nuclear fraction was significantly increased, while the expression in the cytoplasmic fraction was decreased compared to the control group. These results indicated that exposure to PM_2.5_ could cause ROS formation and activate NRF2 to defense oxidative stress.

### PM_2.5_ Inhibits Energy Metabolism and Thus Results in Deficiencies of Amino Acids and Nucleotides in GC-2spd

**Figure 5:**
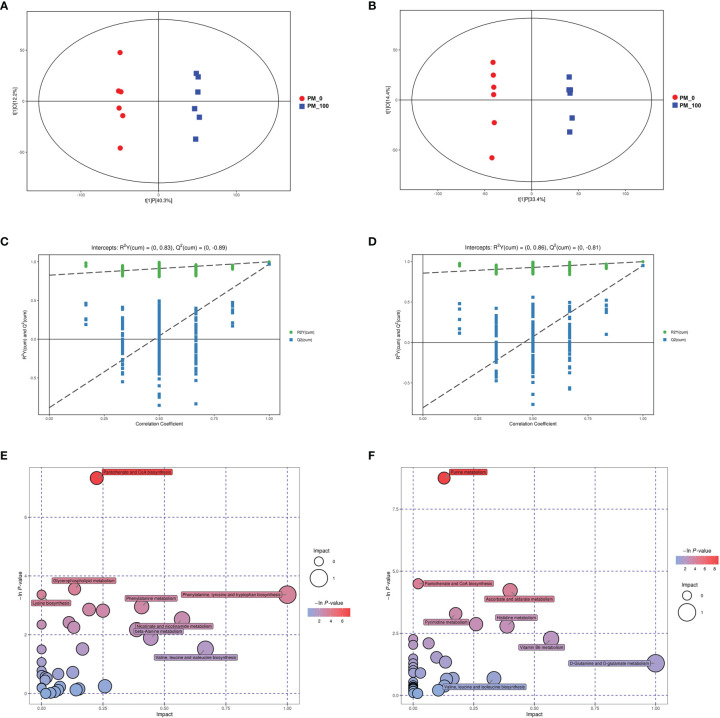
Changes in metabolic profile in GC-2spd after exposure to PM_2.5_ (0, 100 μg/mL) for 48 h. The OPLS–DA model **(A, B)** and permutation test of the OPLS–DA model **(C, D)** were derived from the LC/MS metabolomics profiles. **(E, F)** Analysis of the metabolic pathways of GC-2spd exposed to PM_2.5_ are shown. Each circle represents a metabolic pathway, and the size of the circle indicates the relative impact value. **(A, C, E)** were derived from the positive ionization mode, and **(B, D, F)** were derived from the negative ionization mode.

Energy metabolism is the process by which ATP is generated *via* oxidative phosphorylation in mitochondria (32). We confirmed PM_2.5_ exposure could induce mitochondrial damage and disturb mitochondrial respiration. To further investigate whether PM_2.5_ could affect energy metabolism, metabolomics analysis was conducted to estimate the metabolic variance induced by PM_2.5_ exposure. As shown in **Figures 5A, B**, in the OPLS-DA score plot, the PM_2.5_ exposure group was clearly separated from the control group in both positive and negative ionization modes. The R^2^ and Q^2^ were 0.83 and 0.89 in the positive ionization mode, and 0.86 and 0.81 in the negative ionization mode, respectively (**Figures 5C, D**). These results indicated that the OPLS-DA model had good stability and no overfitting phenomenon. Between the control and PM_2.5_ group, 317 and 128 significantly differential metabolites (SDMs) (*P*<0.05 and VIP>1, one-way ANOVA) were identified in the positive and negative ionization modes, respectively. In addition, the altered metabolites were mainly involved in purine metabolism, pantothenate and CoA biosynthesis, glycerophospholipid metabolism, ascorbate and aldarate metabolism, pyrimidine metabolism and amino acids metabolism (**Figures 5E, F**).

**Figure 6:**
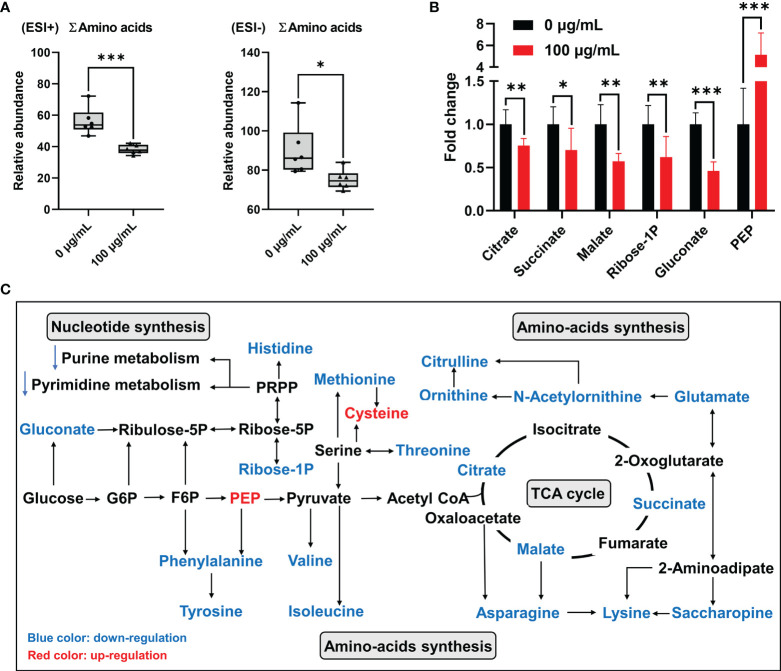
Disorder of energy metabolism and amino acids synthesis in GC-2spd after exposure to PM_2.5_ (0, 100 μg/mL) for 48 h. **(A)** Total concentrations of amino acids (∑amino acids). ESI+ indicates the positive ionization mode, and ESI- indicates the negative ionization mode. **(B)** PM_2.5_-induced changes of the metabolic biomarkers related to central carbon metabolism. **(C)** Most relevant metabolites perturbed by PM_2.5_ in central carbon metabolism and amino acids synthesis. G6P, glucose 6-phosphate; F6P, fructose 6-phosphate; PEP, phosphoenolpyruvate. *P < 0.05, **P < 0.01, and ***P < 0.001. Values are presented as means ± SD (n = 6).

Results of metabolomic analyses indicated that concentrations of total amino acids (∑amino acids) in GC-2spd exposed to PM_2.5_ were significantly decreased by more than 30% and 15% in the positive and negative ionization modes, respectively (**Figure 6A**). Saccharopine was decreased by almost 70% compared to the control group. In addition, L-Histidine, S-Adenosylmethionine, L-Asparagine, L-Lysine, Citrulline, L-Methionine, L-Phenylalanine, L-Isoleucine, L-Valine were decreased by more than 30% compared to the control group. More detailed information was listed in **Supplemental Table 1**.

Among these significantly decreased amino acids, some of them were involved in central carbon metabolism, especially the citrate cycle (TCA cycle). Furthermore, citrate, succinate and malate in the TCA cycle were significantly decreased by 25%, 30% and 43% in GC-2spd exposed to PM_2.5_, respectively (**Figures 6B, C**). These results suggested that PM_2.5_ exposure suppressed the central hub of oxidative metabolism, and then affected amino-acids synthesis. Besides, in central carbon metabolism, pyruvate was associated with valine, leucine and isoleucine biosynthesis and glycine, serine and threonine metabolism (**Figure 6C**). Pentose phosphate pathway (PPP) is a way of oxidative decomposition of glucose, which provides variety of raw materials for synthesis and metabolism, like nucleotide synthesis and histidine metabolism (**Figure 6C**). The concentration of glucose 6-phosphosphate (G6P) had no significant change, however, gluconate, which converted from glucose was involved in PPP and significantly decreased compared to the control group (**Figure 6B**).

**Figure 7:**
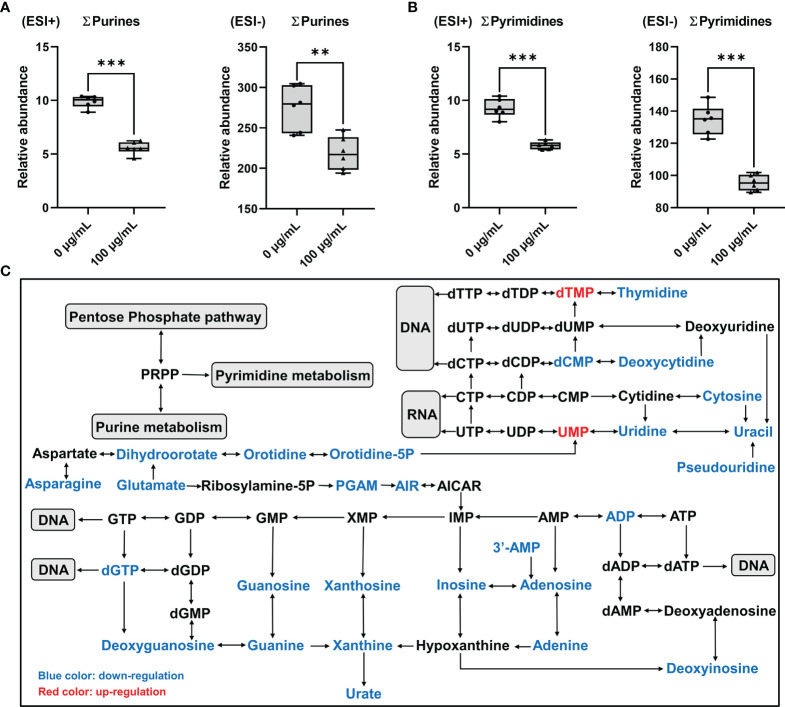
Disorder of nucleotide metabolism in GC-2spd after exposure to PM_2.5_ (0, 100 μg/mL) for 48 h. **(A)** Total concentrations of purines (∑purines). **(B)** Total concentrations of pyrimidines (∑pyrimidines). ESI+ indicates the positive ionization mode, and ESI- indicates the negative ionization mode. **(C)** Most relevant metabolites perturbed by PM_2.5_ in purine metabolism and pyrimidine metabolism. **P < 0.01 and ***P < 0.001. Values are presented as means ± SD (n = 6).

Total concentrations of purines (∑purines) and pyrimidines (∑pyrimidines) were significantly decreased by more than 20% and 29% in GC-2spd exposed to PM_2.5_, respectively (**Figures 7A, B**), which indicated that exposure to PM_2.5_ could disrupt nucleotide synthesis. In purine metabolism, concentrations of dGTP, Guanosine, ADP, deoxyguanosine, deoxyinosine, adenosine and hypoxanthine were significantly decreased by more than 50% compared to the controls (**Supplemental Table 2**). In pyrimidine metabolism, concentrations of thymidine, thymine, deoxycytidine and dCMP were all 50% less than that in GC-2spd exposed to PM_2.5_ (**Supplemental Table 3**). In addition, except for PPP, asparagine and glutamate were involved in purine and pyrimidine metabolism and were significantly decreased by more than 40% and 10% compared to the controls, respectively (**Figure 7C** and **Supplemental Table 1**).

## Discussion

In the present study, we found PM_2.5_ induced the reduction of sperm motility and spermatocyte mitochondrial damage using the real time whole-body PM_2.5_ exposure mouse model. To investigate the potential mechanism of male reproductive toxicity, the spermatocyte-derived GC-2spd was treated with PM_2.5_. Our results indicated that PM_2.5_ exposure inhibited GC-2spd cell proliferation and induced mitochondrial damage. Besides, increased Humanin and MOTS-c levels and decreased mitochondrial respiratory indicated that mitochondrial function was disturbed. Nontargeted metabolomics revealed that PM_2.5_ exposure inhibited energy metabolism and thus resulted in the deficiencies of amino acids and nucleotides. On the other hand, The AhR pathway was activated and resulted in an increase in CYP1A1 expression, which was also observed in CAP-exposed mice. The transcriptomics also confirmed that *Cyp1a1* was the most significantly changed gene. Furthermore, exposure to PM_2.5_ promoted ROS generation and activated NRF2 antioxidative pathway. These findings provided new insights into understanding the mechanisms of PM_2.5_-induced male reproductive toxicity.

Sperm motility is one of the most important sperm parameters to evaluate male fertility (33). Our data demonstrated that exposure to CAP significantly reduced sperm motility, which is consistent with previous studies *via* the whole-body PM exposure model (27). Thus, the present study provided more substantial evidence for the reproductive toxicity by exposure to PM_2.5_. Whereas, no significant change in sperm concentration was found in our study. Zhou et al. (27). and Li et al. (34). obtained the same effect when exposed for 8 weeks. But when the exposure time was extended to 4 months, there was a significant reduction in sperm concentration (27, 35, 36). Up to now, few researches explored the mechanism of reproductive toxicity reduced by PM_2.5_ applying cell lines *in vitro*. Previous studies indicated that PM_2.5_ could inhibit GC-2spd cell proliferation by ROS caused-DNA damage and induce cell apoptosis by RIPK1 and mitochondrial apoptosis pathways (11, 24). Similarly, our present results demonstrated that exposure to PM_2.5_ could inhibit cell proliferation and lead to mitochondrial damage. What’s more, we investigated two possible toxicological mechanisms for the first time including 1) PM_2.5_ exposure might induce energy metabolism disorder by inhibiting mitochondrial respiratory, and thus result in the deficiencies of amino acids and nucleotides; 2) the AhR-CYP1A1 pathway was activated and might cause ROS generation after exposure to PM_2.5_.

Energy metabolism is the process of ATP generation through oxidative phosphorylation and glycolysis. In the present study, exposure to PM_2.5_ reduced the concentrations of succinate, citrate and malate, which indicated inhibition of the TCA cycle. The decreased concentration of succinate and inhibition of the TCA cycle would unavoidably reduce the efficiency of electron transfer to ubiquinone, and thus inhibit energy metabolism from oxidative phosphorylation (32). In mice testes, oxidative phosphorylation occurs in mitochondria, and PM_2.5_ restrained oxidative phosphorylation implies that the mitochondrion is an important target of PM_2.5_. In the present study, the observation of mitochondrial ultrastructure in GC-2spd and mice testes indicated that PM_2.5_ exposure-induced mitochondrial damage. Moreover, we conducted Cell Mito Stress Test, which is a standard assay for measuring mitochondrial function in cells, to evaluate the mitochondrial respiratory. The results showed that a decrease in OCR was found, which was a clear indication of mitochondrial dysfunction. In addition, the reductions of basal respiration, maximal respiration, spare capacity, proton leak and ATP production indicated exposure to PM_2.5_ could induce mitochondrial damage, reduce the capability of the cell to respond to an energetic demand and partly affect the mitochondrial ATP production. These findings supported the evidence of mitochondrial dysfunction and energy metabolism disorder induced by PM_2.5_ exposure. As a result of the inhibition of energy metabolism, the active transport of nutrients such as amino acids and nucleotides would inevitably be reduced, which might inhibit various biological reactions and ultimately affect cell growth. In our study, the concentrations of ∑amino acids, ∑pyrimidines and ∑purines were all significantly decreased in GC-2spd exposed to PM_2.5_.

PAHs are well-known agonists of the AhR, a ligand-activated transcription factor in responding to environmental stress *via* regulating the expression of a diverse range of genes, especially monooxygenases cytochrome P450 (CYP) 1A1 and CYP1A2 (16). Previous studies reported that the SRM 1648a PM_2.5_ contained abundant PAHs according to the research report from NIST (26, 37). In the present study, we confirmed that PM_2.5_ exposure could activate AhR in GC-2spd and significantly promote the expression of CYP1A1 in GC-2spd and mice testes. Moreover, the results of RNA-seq analysis provided support to that PM_2.5_-induced spermatocyte damage involved in the activation of AhR-CYP1A1 pathway. In addition, previous studies demonstrated that AhR-dependent production of CYP1A1 was a major source of ROS generation (16, 38). After binding to AhR, PAHs are oxidized by CYPs into epoxides and phenolic intermediates, which are further processed by phase II enzymes. The resulting 3,6‐quinone undergoes redox cycles with generation of ROS and leads to oxidative stress (39, 40). Consistently, our data indicated that exposure to PM_2.5_ induced ROS excessive production in GC-2spd and the NRF2 antioxidative pathway was activated in GC-2spd and mice testes. It is well-known that mitochondria are both the major site for ROS generation and the main target organelle of ROS attack. The present study showed that mitochondrial damage occurred both *in vivo* and *in vitro* study after exposure to PM_2.5_. These results prompted that the activation of AhR-CYP1A1 pathway followed by ROS excessive generation might provide an explanation of mitochondrial damage and dysfunction. Additional, previous studies revealed that PM_2.5_-induced ROS generation may induce spermatogenesis dysfunction *via* ROS-mediated MAPK signaling pathway or autophagy (41–43), which prompted that further studies should be performed to explore the potential interaction between AhR and MAPK signaling pathway, as well as autophagy in PM_2.5_-induced reproductive damage.

The present study provided compelling evidence that PM_2.5_ exposure-induced sperm motility decline and spermatocyte damage. Although we only provided preliminary data in this study, it has several advantages: We investigated the reproductive toxicity of PM_2.5_ exposure *in vivo* and *in vitro*. In animal experiment, a whole-body PM_2.5_ exposure mouse model was established to simulate exposure environment of humans in the real world. Furthermore, multiple omics techniques were applied to evaluate comprehensive biological changes in GC-2spd following exposure to PM_2.5_. The nontargeted metabolomics confirmed that PM_2.5_ exposure inhibited energy metabolism and thus resulted in the deficiencies of amino acids and nucleotides. And the results of transcriptomics raised the importance of the role of AhR-CYP1A1 pathway to PM_2.5_-induced reproductive toxicity. However, there are also some limitations in this study: To further confirm agonism of PM_2.5_ to AhR, expression or knockdown experiments should be conducted to make the conclusion more convincing. In addition, PM_2.5_ is a very complex mixture. The substances that pass through the blood-testis barrier and play a role in male reproductive toxicity are mainly metabolites of toxic components. Previous study has shown that different methods of extraction of PM_2.5_ can lead to different toxic effect (44). Thus, the method of PM_2.5_ extraction and component identification should be considered in future study.

## Conclusions

Overall, in the present study, we used GC-2spd cell line and a real time whole-body PM_2.5_ exposure mouse model to examine PM_2.5_-induced male reproductive toxicity *in vitro* and *in vivo*. Our results indicated that PM_2.5_ exposure inhibited spermatocyte cell proliferation and reduced sperm motility. The reproductive toxicity might be partly explained by energy metabolism disorder induced by mitochondrial damage and dysfunction. The mechanism of mitochondrial damage might be through the AhR-CYP1A1 pathway which resulted in ROS production.

## Data Availability Statement

The datasets presented in this study can be found in online repositories. The names of the repository/repositories and accession number(s) can be found below: https://www.ncbi.nlm.nih.gov/geo/query/acc.cgi?acc=GSE189187.

## Ethics Statement

The animal study was reviewed and approved by Ethics Council of the Army Medical University.

## Author Contributions

FS undertook the study, collected the data, analyzed the data, and drafted the manuscript. ZZ, JW, YW, JD, and YZ helped with the undertaking of the animal study. PZ, XL, FH, and JL helped with the analysis of omics data. LA and JC revised the manuscript, were responsible for the experimental design, and supervised the study. All authors contributed to the article and approved the submitted version.

## Funding

This work was supported by the Key Program of the National Natural Science Foundation of China (No. 81630087), the National Natural Science Foundation of China (No. 82073590) and the National Key Research and Development Program of China (No. 2017YFC1002001).

## Conflict of Interest

The authors declare that the research was conducted in the absence of any commercial or financial relationships that could be construed as a potential conflict of interest.

## Publisher’s Note

All claims expressed in this article are solely those of the authors and do not necessarily represent those of their affiliated organizations, or those of the publisher, the editors and the reviewers. Any product that may be evaluated in this article, or claim that may be made by its manufacturer, is not guaranteed or endorsed by the publisher.
