# Supplementary material for: Analysis by Metabolomics and Transcriptomics for the Energy Metabolism Disorder and the Aryl Hydrocarbon Receptor Activation in Male Reproduction of Mice and GC-2spd Cells Exposed to PM2.5
Source: Front Endocrinol (Lausanne). 2022 Jan 3;12:807374. doi: 10.3389/fendo.2021.807374 (PMC8761788; doi:10.3389/fendo.2021.807374)
Supplement: Supplementary file 1 [file DataSheet_1.docx]

Supplementary Table 1 Identified metabolites involved in amino acid metabolism in GC-2spd after exposure to PM_2.5_ (0, 100 μg/mL) for 48 h.

| Ionisation mode | Metabolites | Mean_100 | Mean_0 | VIP | *P* value | Foldchange | Regulation |
| --- | --- | --- | --- | --- | --- | --- | --- |
| ESI+ | Saccharopine | 0.0041 | 0.0128 | 1.3911 | 0.0000 | 0.32 | Down-regulation |
| ESI+ | S-Adenosylmethionine | 0.0192 | 0.0476 | 1.4445 | 0.0001 | 0.40 | Down-regulation |
| ESI+ | Citrulline | 0.3025 | 0.4700 | 1.4451 | 0.0001 | 0.64 | Down-regulation |
| ESI+ | L-Threonine | 7.2262 | 8.9384 | 1.4013 | 0.0004 | 0.81 | Down-regulation |
| ESI+ | Ornithine | 0.0230 | 0.0303 | 1.3442 | 0.0005 | 0.76 | Down-regulation |
| ESI+ | L-Isoleucine | 5.8275 | 8.7065 | 1.3876 | 0.0006 | 0.67 | Down-regulation |
| ESI+ | L-Valine | 5.5468 | 8.1498 | 1.3946 | 0.0007 | 0.68 | Down-regulation |
| ESI+ | Argininosuccinic acid | 0.0539 | 0.0305 | 1.2507 | 0.0017 | 1.77 | Up-regulation |
| ESI+ | L-Histidine | 0.4554 | 1.1443 | 1.2266 | 0.0038 | 0.40 | Down-regulation |
| ESI+ | L-Phenylalanine | 9.2022 | 13.9906 | 1.3242 | 0.0049 | 0.66 | Down-regulation |
| ESI+ | L-Lysine | 2.6648 | 4.7138 | 1.4026 | 0.0053 | 0.57 | Down-regulation |
| ESI+ | Citric acid | 0.0084 | 0.0111 | 1.1460 | 0.0079 | 0.76 | Down-regulation |
| ESI+ | L-Asparagine | 0.7118 | 1.3827 | 1.3638 | 0.0080 | 0.51 | Down-regulation |
| ESI+ | S-Adenosylhomocysteine | 0.0772 | 0.1060 | 1.0903 | 0.0102 | 0.73 | Down-regulation |
| ESI+ | L-Tyrosine | 2.0225 | 2.8249 | 1.2043 | 0.0128 | 0.72 | Down-regulation |
| ESI+ | N-Acetylornithine | 0.0455 | 0.0570 | 1.0099 | 0.0186 | 0.80 | Down-regulation |
| ESI+ | L-Methionine | 3.2052 | 4.8814 | 1.0985 | 0.0252 | 0.66 | Down-regulation |
| ESI+ | L-Glutamic acid | 0.3053 | 0.2127 | 0.5117 | 0.1069 | 1.44 | Up-regulation |
| ESI+ | L-Tryptophan | 0.0040 | 0.0032 | 0.3806 | 0.1650 | 1.28 | Up-regulation |
| ESI+ | L-Glutamine | 0.4582 | 0.4881 | 0.4861 | 0.1768 | 0.94 | Down-regulation |
| ESI- | L-Threonine | 13.2610 | 16.1757 | 1.3910 | 0.0048 | 0.82 | Down-regulation |
| ESI- | Phosphoenolpyruvic acid | 0.5602 | 0.1089 | 1.5690 | 0.0065 | 5.15 | Up-regulation |
| ESI- | N-Acetylornithine | 0.0378 | 0.0535 | 1.2995 | 0.0075 | 0.71 | Down-regulation |
| ESI- | L-Cysteine | 0.4486 | 0.0194 | 1.6332 | 0.0135 | 23.11 | Up-regulation |
| ESI- | L-Histidine | 0.8880 | 1.7023 | 1.4850 | 0.0190 | 0.52 | Down-regulation |
| ESI- | L-Valine | 5.5264 | 7.9740 | 1.3608 | 0.0212 | 0.69 | Down-regulation |
| ESI- | L-Glutamic acid | 32.5151 | 37.3474 | 1.1858 | 0.0216 | 0.87 | Down-regulation |
| ESI- | L-Lysine | 0.6816 | 1.1251 | 1.3988 | 0.0262 | 0.61 | Down-regulation |
| ESI- | L-Asparagine | 1.4098 | 2.4074 | 1.3289 | 0.0274 | 0.59 | Down-regulation |
| ESI- | L-Arginine | 0.7395 | 1.0741 | 1.2073 | 0.0568 | 0.69 | Down-regulation |
| ESI- | L-Phenylalanine | 5.9339 | 8.1924 | 1.0509 | 0.0648 | 0.72 | Down-regulation |
| ESI- | S-Adenosylhomocysteine | 0.0321 | 0.0391 | 0.9055 | 0.0828 | 0.82 | Down-regulation |
| ESI- | 2-Phospho-D-glyceric acid | 0.5602 | 0.3332 | 0.8315 | 0.1020 | 1.68 | Up-regulation |
| ESI- | L-Tyrosine | 2.2237 | 2.7660 | 0.8971 | 0.1061 | 0.80 | Down-regulation |
| ESI- | L-Serine | 5.0961 | 5.7066 | 0.7075 | 0.1616 | 0.89 | Down-regulation |
| ESI- | L-Isoleucine | 0.1519 | 0.1906 | 0.5657 | 0.1758 | 0.80 | Down-regulation |
| ESI- | Citrulline | 0.0053 | 0.0181 | 0.8867 | 0.1805 | 0.29 | Down-regulation |
| ESI- | Citric acid | 0.2447 | 0.3834 | 0.5084 | 0.1823 | 0.64 | Down-regulation |
| ESI- | L-Proline | 0.0887 | 0.1195 | 0.3368 | 0.2704 | 0.74 | Down-regulation |
| ESI- | Pyruvic acid | 4.6691 | 4.2316 | 0.1269 | 0.3408 | 1.10 | Up-regulation |
| ESI- | D-Erythrose 4-phosphate | 0.1279 | 0.1353 | 0.2397 | 0.3465 | 0.95 | Down-regulation |

Supplementary Table 2 Identified metabolites involved in purine metabolism in GC-2spd after exposure to PM_2.5_ (0, 100 μg/mL) for 48 h.

| Ionisation mode | Metabolites | Mean_100 | Mean_0 | VIP | *P* value | Foldchange | Regulation |
| --- | --- | --- | --- | --- | --- | --- | --- |
| ESI+ | Guanosine | 0.7281 | 1.8585 | 1.5643 | 0.0000 | 0.39 | Down-regulation |
| ESI+ | Inosine | 1.1914 | 2.2327 | 1.4702 | 0.0000 | 0.53 | Down-regulation |
| ESI+ | Deoxyinosine | 0.4017 | 0.9624 | 1.4610 | 0.0000 | 0.42 | Down-regulation |
| ESI+ | Deoxyguanosine | 0.3595 | 0.7892 | 1.4321 | 0.0001 | 0.46 | Down-regulation |
| ESI+ | 5-Aminoimidazole ribonucleotide | 0.5026 | 0.8085 | 1.4182 | 0.0002 | 0.62 | Down-regulation |
| ESI+ | Cyclic AMP | 0.0023 | 0.0045 | 1.4357 | 0.0016 | 0.51 | Down-regulation |
| ESI+ | Hypoxanthine | 0.3143 | 1.0032 | 1.2393 | 0.0022 | 0.31 | Down-regulation |
| ESI+ | Adenosine 3',5'-diphosphate | 0.0397 | 0.1093 | 1.2384 | 0.0030 | 0.36 | Down-regulation |
| ESI+ | Xanthosine | 0.0046 | 0.0071 | 1.0466 | 0.0158 | 0.65 | Down-regulation |
| ESI+ | Xanthine | 0.0203 | 0.0310 | 1.0555 | 0.0205 | 0.65 | Down-regulation |
| ESI+ | Inosinic acid | 0.1079 | 0.0497 | 0.7842 | 0.0715 | 2.17 | Up-regulation |
| ESI+ | Deoxyadenosine | 0.1331 | 0.2006 | 0.7430 | 0.0715 | 0.66 | Down-regulation |
| ESI+ | Adenine | 0.8395 | 0.8998 | 0.7589 | 0.0724 | 0.93 | Down-regulation |
| ESI+ | Allantoic acid | 0.0108 | 0.0148 | 0.5143 | 0.1244 | 0.73 | Down-regulation |
| ESI+ | L-Glutamine | 0.4582 | 0.4881 | 0.4861 | 0.1768 | 0.94 | Down-regulation |
| ESI+ | Adenosine monophosphate | 0.0196 | 0.0169 | 0.1420 | 0.2452 | 1.16 | Up-regulation |
| ESI+ | Guanosine diphosphate | 0.0024 | 0.0025 | 0.0666 | 0.2518 | 0.95 | Down-regulation |
| ESI+ | Guanine | 0.0815 | 0.0777 | 0.3096 | 0.2598 | 1.05 | Up-regulation |
| ESI+ | Adenosine | 0.2928 | 0.2781 | 0.0605 | 0.2756 | 1.05 | Up-regulation |
| ESI+ | Guanosine monophosphate | 0.0513 | 0.0520 | 0.1829 | 0.2970 | 0.99 | Down-regulation |
| ESI- | Guanosine | 1.2571 | 3.1835 | 1.7004 | 0.0000 | 0.39 | Down-regulation |
| ESI- | Adenosine | 0.0163 | 0.0334 | 1.5745 | 0.0000 | 0.49 | Down-regulation |
| ESI- | Deoxyinosine | 26.1397 | 55.6834 | 1.6044 | 0.0001 | 0.47 | Down-regulation |
| ESI- | Inosine | 7.3282 | 12.9549 | 1.5576 | 0.0002 | 0.57 | Down-regulation |
| ESI- | Adenine | 0.6368 | 0.9719 | 1.5431 | 0.0003 | 0.66 | Down-regulation |
| ESI- | Deoxyguanosine | 1.1599 | 2.6117 | 1.5277 | 0.0006 | 0.44 | Down-regulation |
| ESI- | Xanthine | 10.7729 | 15.9184 | 1.5544 | 0.0052 | 0.68 | Down-regulation |
| ESI- | dGTP | 0.0047 | 0.0131 | 1.4618 | 0.0070 | 0.36 | Down-regulation |
| ESI- | Uric acid | 0.0762 | 0.1175 | 1.2530 | 0.0078 | 0.65 | Down-regulation |
| ESI- | Cyclic AMP | 0.0018 | 0.0034 | 1.3617 | 0.0094 | 0.55 | Down-regulation |
| ESI- | ADP | 0.0558 | 0.1263 | 1.2369 | 0.0162 | 0.44 | Down-regulation |
| ESI- | Guanine | 3.4118 | 4.7498 | 1.1998 | 0.0260 | 0.72 | Down-regulation |
| ESI- | Xanthosine | 0.0429 | 0.0620 | 1.1502 | 0.0394 | 0.69 | Down-regulation |
| ESI- | Inosinic acid | 0.2964 | 0.1238 | 1.0070 | 0.0868 | 2.39 | Up-regulation |
| ESI- | Deoxyadenosine monophosphate | 0.4042 | 0.3818 | 0.6794 | 0.1561 | 1.06 | Up-regulation |
| ESI- | Hypoxanthine | 165.7601 | 177.3247 | 0.5998 | 0.1777 | 0.93 | Down-regulation |
| ESI- | Guanosine monophosphate | 0.0769 | 0.0653 | 0.2839 | 0.2253 | 1.18 | Up-regulation |
| ESI- | Adenosine monophosphate | 0.7955 | 0.6524 | 0.1437 | 0.3208 | 1.22 | Up-regulation |
| ESI- | Deoxyadenosine | 0.1311 | 0.1552 | 0.3367 | 0.3466 | 0.84 | Down-regulation |
| ESI- | dIMP | 0.0128 | 0.0127 | 0.0548 | 0.4052 | 1.01 | Up-regulation |
| ESI- | Adenylsuccinic acid | 0.0059 | 0.0057 | 0.1796 | 0.4083 | 1.04 | Up-regulation |

Supplementary Table 3 Identified metabolites involved in pyrimidine metabolism in GC-2spd after exposure to PM_2.5_ (0, 100 μg/mL) for 48 h.

| Ionisation mode | Metabolites | Mean_100 | Mean_0 | VIP | *P* value | Foldchange | Regulation |
| --- | --- | --- | --- | --- | --- | --- | --- |
| ESI+ | Deoxycytidine | 0.1748 | 0.3815 | 1.5455 | 0.0000 | 0.46 | Down-regulation |
| ESI+ | dCMP | 0.2453 | 0.5345 | 1.5374 | 0.0000 | 0.46 | Down-regulation |
| ESI+ | Thymidine | 0.0441 | 0.1186 | 1.4649 | 0.0000 | 0.37 | Down-regulation |
| ESI+ | Thymine | 1.0016 | 2.6010 | 1.4592 | 0.0001 | 0.39 | Down-regulation |
| ESI+ | Uridine | 0.4428 | 0.6130 | 1.3982 | 0.0004 | 0.72 | Down-regulation |
| ESI+ | Uracil | 2.9293 | 4.0832 | 1.3957 | 0.0004 | 0.72 | Down-regulation |
| ESI+ | Uridine 5'-monophosphate | 0.1513 | 0.0925 | 0.9054 | 0.0347 | 1.64 | Up-regulation |
| ESI+ | Cytidine monophosphate | 0.0738 | 0.0426 | 0.7117 | 0.0636 | 1.73 | Up-regulation |
| ESI+ | Dihydrothymine | 0.2452 | 0.2928 | 0.7071 | 0.0866 | 0.84 | Down-regulation |
| ESI+ | Dihydrouracil | 0.0268 | 0.0294 | 0.4375 | 0.1397 | 0.91 | Down-regulation |
| ESI+ | L-Glutamine | 0.4582 | 0.4881 | 0.4861 | 0.1768 | 0.94 | Down-regulation |
| ESI- | dCMP | 0.6751 | 1.2723 | 1.6767 | 0.0000 | 0.53 | Down-regulation |
| ESI- | Thymine | 1.5823 | 2.4591 | 1.6576 | 0.0000 | 0.64 | Down-regulation |
| ESI- | Thymidine | 35.5462 | 60.8639 | 1.6589 | 0.0000 | 0.58 | Down-regulation |
| ESI- | Deoxycytidine | 0.1489 | 0.4938 | 1.6803 | 0.0001 | 0.30 | Down-regulation |
| ESI- | 4,5-Dihydroorotic acid | 0.0513 | 0.0652 | 1.4788 | 0.0014 | 0.79 | Down-regulation |
| ESI- | Orotic acid | 0.0915 | 0.1844 | 1.4893 | 0.0015 | 0.50 | Down-regulation |
| ESI- | Uridine | 29.9084 | 36.8991 | 1.4631 | 0.0015 | 0.81 | Down-regulation |
| ESI- | Uridine 5'-monophosphate | 0.1683 | 0.1304 | 1.3846 | 0.0037 | 1.29 | Up-regulation |
| ESI- | Uracil | 26.6834 | 31.6671 | 1.2959 | 0.0223 | 0.84 | Down-regulation |
| ESI- | 5-Thymidylic acid | 0.2505 | 0.2057 | 1.0362 | 0.0414 | 1.22 | Up-regulation |
| ESI- | Cytidine monophosphate | 0.1172 | 0.0572 | 1.0546 | 0.0671 | 2.05 | Up-regulation |
| ESI- | Cytidine | 0.1561 | 0.1303 | 0.3699 | 0.2481 | 1.20 | Up-regulation |
| ESI- | Deoxyuridine | 0.0555 | 0.0595 | 0.3063 | 0.2728 | 0.93 | Down-regulation |
| ESI- | 3-Aminoisobutanoic acid | 0.0714 | 0.0730 | 0.2664 | 0.3204 | 0.98 | Down-regulation |
